# Disparities in All-Cause Mortality in Older Patients with Colorectal Cancer According to Disability Status: A Nationwide Analysis

**DOI:** 10.3390/curroncol29100584

**Published:** 2022-10-05

**Authors:** Woo-Ri Lee, Kyu-Tae Han, Mingee Choi, Woorim Kim

**Affiliations:** 1 Division of Cancer Control & Policy, National Cancer Control Institute, National Cancer Center, Goyang-si 10408, Korea; 2 Interdisciplinary Graduate Program in Social Welfare Policy, Yonsei Graduate School, Yonsei University, Seodaemun-gu, Seoul 03722, Korea

**Keywords:** colorectal cancer, disability, mortality, survival, older patients

## Abstract

Background: Although investigating patterns of cancer mortality is important in understanding the effect of cancer on population health, knowledge regarding mortality in cancer patients with disability is scarce. This study examined the association between disability status and all-cause mortality in older patients with colorectal cancer. Methods: Data were obtained from the 2008–2019 National Health Insurance Service claims data. The study population included patients with colorectal cancer aged 60 years or above. The outcome measure was all-cause 5-year and overall mortality. A survival analysis was performed using the Cox proportional hazards model to analyze the association between all-cause mortality and disability status. Subgroup analysis was conducted based on disability severity. Results: The study population consisted of 6340 patients, and disability was reported in 15.8% of the included individuals. Participants with disability had a higher risk of both all-cause 5-year (hazard ratio (HR) 1.21, 95% confidence interval (95% CI) 1.07–1.37) and overall mortality (HR 1.15, 95% CI 1.03–1.28). These findings were particularly significant in individuals with severe rather than mild disability. Conclusion: Older colorectal cancer patients with disabilities showed a higher risk of overall and 5-year all-cause mortality, which was evident in individuals with severe disabilities. The findings indicated disparities in mortality according to disability status. Further, we suggest that policies that can mediate such disparities must be strengthened.

## 1. Introduction

Colorectal cancer (CRC) constitutes a noticeable global public health burden, as it is the third most common and second most deadly type of cancer worldwide, with an estimated incidence of approximately 1.9 million cases in 2020 [1]. The number of cases of CRC is also escalating in many Asian countries including South Korea, which is partially influenced by a westernized diet, reduced physical activity, alcohol consumption, and increased body mass index (BMI) [2]. Currently, CRC is the second leading cancer in Korea and ranks third highest with respect to the number of deaths caused due to cancer [3,4]. In response to the increasing burden of cancer, Korea has implemented a national cancer screening program that includes CRC as one of its target cancer types. This has contributed to early detection of CRC in the Korean population [5]. Yet, the burden of CRC persists. Furthermore, as Korea is an aging country, constant monitoring is required. CRC is often reported as a disease of the elderly, and age is regarded as a major risk factor [2,6].

Understanding patterns of cancer mortality can provide an insight into the effect of cancer on population health, and has hence been mostly well documented. However, knowledge regarding differences in mortality in CRC patients with disability is largely deficient despite disabled individuals representing a major group of vulnerable populations, given that approximately 15% of the global population has disabilities [7]. The number of people with disabilities has also increased in Korea from approximately 2.1 million in 2005 to 2.7 million in 2017 [8]. Investigating mortality in cancer patients with disability is important because cancer is usually detected at a later stage in such populations and patients show poorer survival [9,10]. Individuals with disabilities are known to experience various barriers, including physical and communicational constraints, in accessing and utilizing healthcare [11,12,13]. People with disabilities also often have lower education or income, which may lead to further disparities in cancer diagnosis, treatment, and prognosis [9,14].

Disability not only impedes independence but also increases dependence on family or social support for survival, which leads to an increased social burden and a decreased quality of life, especially in the elderly. Therefore, investigating potential differences in mortality according to disability status in older patients with CRC [15] is required. The objective of this study was to examine the association of disability status with overall and 5-year all-cause mortality in patients with CRC aged 60 years or above. The hypothesis was that CRC patients with disabilities would have a higher risk of mortality than those without disabilities. Additional subgroup analysis was conducted based on disability severity.

## 2. Methods

### 2.1. Data and Study Population

This study used data from the 2008–2019 National Health Insurance Service (NHIS) senior cohort. This cohort includes claims data from approximately 8% of the entire population aged from 60 to 80 years in 2008 that was selected through random sampling after stratification based on sex, age, the level of health insurance premium, and region [16]. The study population consisted of individuals who were first diagnosed with CRC (International Classification of Diseases [ICD]-10: C18–C21) at age 60 years or above (N = 11,496). Subsequently, 5156 individuals who did not receive any type of treatment for CRC within 6 months of cancer diagnosis or who died within 1 month of cancer diagnosis were excluded. The final study population consisted of 6340 participants.

### 2.2. Variables

The outcome measures of this study were all-cause 5-year and overall mortality. The dates listed for diagnosis of CRC and issuance of the cancer-specific insurance claims code were used to set the index date. Study participants were categorized based on their overall and 5-year survival status after diagnosis.

The primary independent variable was disability status, which was categorized based on the Certificate of Person with Disability distributed by the government [17]. Individuals can be registered as persons with disability after applying and undergoing an examination. Both physical and mental disabilities are included, namely physical impairment, brain impairment, visual impairment, hearing impairment, linguistic impairment, mental impairment, developmental disability, and others (impairment due to renal functioning, heart functioning, respiratory functioning, liver functioning, intestinal functioning, or urinal tract functioning, epilepsy, and facial disfigurement). The severity of disabilities was classified based on the standard used by the disability registration system in Korea, in which the severity of disability ranges from grades one to six, wherein grades one to three are classified as severe disability and four to six as mild disability [18]. The category of mild disability includes individuals who can perform some level of daily tasks with the use of partial personal assistance of assistive devices whereas severe disability refers to those who require heavy dependence on personal assistance or assistive devices [19].

Various covariates were included in the analysis, such as sex (male or female), age (60–69, 70–79, or 80+ years), income (quartiles), type of healthcare insurance (medical aid, NHI self-employed, or NHI employee), region (urban or rural), chronic diseases (none or 1+), level of comorbidity, type of cancer treatment (surgery only, surgery plus chemotherapy or radiotherapy, or surgery plus both chemotherapy and radiotherapy), and type of hospital (tertiary or general hospital). Chronic diseases referred to the presence of diabetes, hypertension, or dyslipidemia that were identified based on ICD-10 codes E10-E14, I10-I15, and E78. The level of comorbidity was measured using the Charlson Comorbidity Index (CCI), which was scored after excluding cancer. The composite CCI score was obtained by summing the weighted score of 17 comorbidities and indicates the level of disease burden [20].

### 2.3. Statistical Analysis

The general characteristics of the study population were examined based on the chi-square test. Kaplan–Meier survival curves and log-rank tests performed based on disability status were used to compare survival time. Proportional hazard assumption was also tested through the Kaplan–Meier survival curve. Survival analysis using the Cox proportional hazards model for overall and 5-year mortality were also conducted after adjusting for all the covariates. Results were expressed in hazard ratios (HR) and their 95 percent confidence intervals (95% CI). Additional subgroup analysis was performed based on disability severity. P-values were two-sided and considered significant at <0.05. All survival analyses were conducted as multivariate analysis after adjusting for all covariates. All statistical analyses were executed using the SAS statistical software, version 9.4 (Cary, NC, USA).

## 3. Results

General characteristics of the study population are presented in Table 1. From 6340 patients with CRC aged 60 years or above, 1733 (27.3%) individuals died within five years of cancer diagnosis and 2334 (36.8%) individuals died within the entire study period. Disability was found in 999 individuals, which accounted for approximately 15.8% of the entire study population. Both 5-year (32.8%) and overall mortality (42.0%) were more frequent in participants with disability.

**Figure 1:**
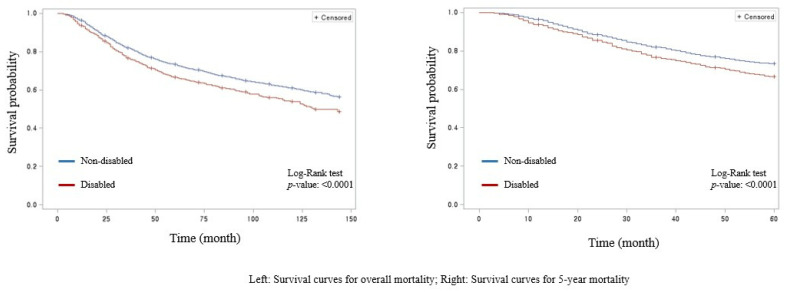
Kaplan–Meier survival curves according to disability status.

**Figure 2:**
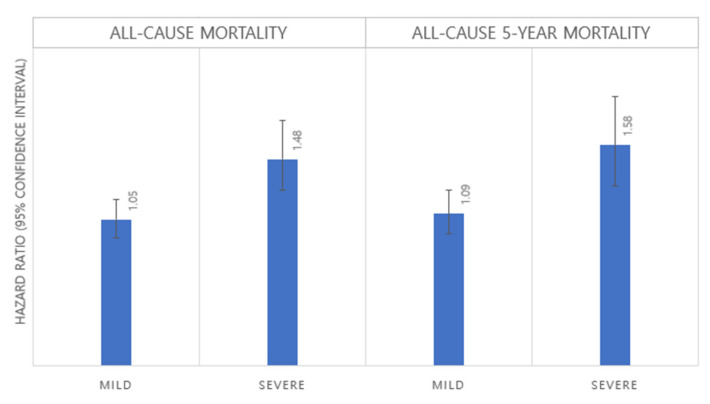
The association between disability status and mortality according to disability severity.

The results of the survival analysis regarding the association between all-cause mortality and disability status are shown in Table 2. Kaplan–Meier survival curves between those with and without disability are found in Figure 1. Participants with disability had a higher risk of both all-cause 5-year (hazard ratio [HR] 1.21, 95% confidence interval [95% CI] 1.07–1.37) and overall mortality (HR 1.15, 95% CI 1.03–1.28). Results of the subgroup analysis based on the severity level of disability are revealed in Table 3 and Figure 2. Tendencies found in the main findings were maintained regardless of the severity of disability, as patients with disability tended to have higher overall and 5-year all-cause mortality. Specifically, these tendencies were particularly pronounced in individuals with severe (5-year mortality: HR 1.58, 95% CI 1.29–1.93; overall mortality: HR 1.48, 95% CI 1.24–1.76) rather than mild (five-year mortality: HR 1.09, 95% CI 0.95–1.26; overall mortality: HR 1.05, 95% CI 0.92–1.19) disability.

## 4. Discussion

This study investigated the effect of disability status on all-cause mortality in patients with CRC aged 60 years or above using nationwide data from Korea. The results revealed that the percentage of individuals who survived overall and for 5 years was higher in cancer patients without disabilities, as compared to those with disabilities. Likewise, older CRC patients with disability also showed a higher risk of overall and 5-year all-cause mortality compared to those without disability. These tendencies were particularly significant in study participants with severe disability. Our findings provide further evidence that disparities in mortality may exist according to disability status in older patients with CRC, although the fact that individuals with disabilities tend to have a generally shorter life expectancy than those without disabilities should be concurrently taken into consideration when interpreting the study results [21].

The study results are in accordance with a previous study, which revealed that cancer patients with disabilities tended to have higher mortality compared to cancer patients without disabilities [22]. For instance, a study regarding Medicare beneficiaries in the United States concluded that disabled beneficiaries diagnosed with breast cancer and CRC had higher overall and cancer-specific mortality. A study of the Dutch adult population also found that cancer-related mortality was more common in individuals with intellectual disability [10]. Likewise, a previous study in Korea reported that cancer patients with disabilities had higher long-term all-cause mortality, and that such propensities also tended to persist in 5-year cancer survivors, suggesting the need for further collaborative efforts to improve the survival of cancer patients and survivors [23]. Another study similarly demonstrated a higher risk of overall mortality in cervical cancer patients with disabilities and concluded that social support and policies were needed to improve such disparities [24]. Lower survival rates were also found in disabled individuals diagnosed with multiple myeloma compared to their non-disabled counterparts [25].

The excessive number of deaths in CRC patients with disabilities may, in part, be because those with disabilities generally have higher comorbidities and face poorer socioeconomic conditions [9]. Complex health conditions underlying certain types of disabilities, combined with the side effects of cancer treatment, may have led to a higher risk of mortality in cancer patients with disabilities [26]. Treatment options for cancer may also be comparatively limited for some patients with disabilities for several reasons, which include difficulties in easily accessing transportation on a daily basis to receive treatment in healthcare institutions [27]. Further, medical institutions may also lack various facilities that allow easy access for patients with disabilities [28]. Patients with disabilities are often less likely to receive treatment for cancer, which may be partially influenced by some medical professionals discouraging treatment or the disabled individuals deciding to give up treatment because of an underestimation of the benefits of treatment or an overestimation of the possibility of treatment-related complications [29].

This study had some limitations. First, cancer severity, including the stage of cancer at diagnosis or pathologic test results, could not be accounted for because of data limitations. However, the analysis excluded individuals who died within 1 month of diagnosis or those who were not hospitalized to receive treatment for cancer in order to enhance homogeneity of the study population. Second, information regarding certain characteristics that may have been important in evaluating mortality after treatment, such as adherence to postoperative care or suitability of the type of oncologic treatment received, were unavailable. Third, this study only considered overall mortality, but not cancer-specific mortality, as its outcomes were variable owing to the limitations in available data. Future studies examining cancer-specific mortality in CRC patients with disabilities are required to develop further understanding in this regard. Last, it has been previously reported that people with disabilities tend to have a shorter life expectancy than the general population and such tendencies may be comparatively pronounced in older individuals [29]. This study only investigated the effect of disabilities on all-cause mortality in patients with CRC. Further studies examining the synergistic effect between disability and CRC on mortality compared to non-disabled individuals are needed to gain further insights. The findings emphasize the importance of developing and strengthening healthcare policies that can reduce disparities in mortality of cancer patients according to their disability status, particularly in older individuals, as many countries face an aging population and cancer occurrence is known to increase with age.

## 5. Conclusions

Patients with CRC aged 60 years or above with disabilities, particularly severe disabilities, showed a higher risk of overall and 5-year all-cause mortality than those without disabilities. The findings reveal the existence of disparities in survival rates according to disability status in older patients with CRC. Efforts are needed to strengthen healthcare policies and guidelines that can reduce disability-related disparities found in this study.

## Figures and Tables

**Table 1:** General characteristics of the study population.

	Total	All-Cause Mortality			All-Cause 5-Year Mortality		
		N	(%)	*p*-Value	N	(%)	*p*-Value
**Disability**							
No	5341	1914	(35.8)	<0.001	1405	(26.3)	<0.001
Yes	999	420	(42.0)		328	(32.8)	
**Disability severity**							
None	5341	1914	(35.8)	0.011	1405	(26.3)	0.003
Mild	714	282	(39.5)		222	(31.1)	
Severe	285	138	(48.4)		106	(37.2)	
**Sex**							
Male	3881	1494	(38.5)	<0.001	1098	(28.3)	0.030
Female	2459	840	(34.2)		635	(25.8)	
**Age**							
60–69	2923	817	(28.0)	<0.001	603	(20.6)	<0.001
70–79	2904	1239	(42.7)		915	(31.5)	
≥80	513	278	(54.2)		215	(41.9)	
**Income**							
Q1	1437	567	(39.5)	0.110	423	(29.4)	0.130
Q2	1111	403	(36.3)		308	(27.7)	
Q3	1750	640	36.6)		474	(27.1)	
Q4	2042	724	(35.5)		528	(25.9)	
**Type of healthcare insurance**							
Medical Aid	355	171	(48.2)	<0.001	125	(35.2)	0.003
NHI Self employed	1831	654	(35.7)		484	(26.4)	
NHI Employee	4154	1509	(36.3)		1124	(27.1)	
**Region**							
Urban	4103	1449	(35.3)	<0.001	1082	(26.4)	0.020
Rural	2237	885	(39.6)		651	(29.1)	
**Chronic diseases**							
None	715	243	(34.0)	0.100	186	(26.0)	0.400
≥1	5625	2091	(37.2)		1547	(27.5)	
CCI *							
0	1391	305	(21.9)	<0.001	194	(13.9)	<0.001
1	896	259	(28.9)		160	(17.9)	
2	806	222	(27.5)		146	(18.1)	
≥3	3247	1548	(47.7)		1233	(38.0)	
**Type of treatment**							
Surgery only	5102	1637	(32.1)	<0.001	1151	(22.6)	<0.001
Surgery and chemo or radiotherapy	788	362	(45.9)		270	(34.3)	
Chemo or radiotherapy only	450	335	(74.4)		312	(69.3)	
**Type of hospital**							
Tertiary hospital	3918	1384	(35.3)	0.002	1017	(26.0)	0.002
General hospital	2422	950	(39.2)		716	(29.6)	
**Total**	**6340**	**2334**	**(36.8)**		**1733**	**(27.3)**	

* CCI: Charlson Comorbidity Index.

**Table 2:** Results of the Cox regression analysis on the association between disability status and mortality.

	All-Cause Mortality			All-Cause 5-Year Mortality		
	HR *	95% CI *		HR *	95% CI *	
**Disability**						
No	1.00			1.00		
Yes	1.15	(1.03	1.28)	1.21	(1.07	1.37)
**Sex**						
Male	1.00			1.00		
Female	0.81	(0.74	0.88)	0.85	(0.77	0.93)
**Age**						
60–69	1.00			1.00		
70–79	1.82	(1.66	1.99)	1.73	(1.55	1.92)
≥80	3.18	(2.76	3.66)	2.91	(2.47	3.42)
**Income**						
Q1	1.00			1.00		
Q2	1.03	(0.90	1.19)	1.05	(0.90	1.23)
Q3	0.99	(0.88	1.13)	0.99	(0.85	1.14)
Q4	0.93	(0.82	1.05)	0.92	(0.79	1.06)
**Type of healthcare insurance**						
Medical Aid	1.00			1.00		
NHI Self employed	0.84	(0.69	1.02)	0.89	(0.71	1.12)
NHI Employee	0.86	(0.71	1.03)	0.91	(0.73	1.12)
**Region**						
Urban	1.00			1.00		
Rural	1.1	(1.01	1.20)	1.07	(0.97	1.19)
**Chronic diseases**						
None	1.00			1.00		
≥1	0.94	(0.82	1.08)	0.87	(0.75	1.02)
CCI *						
0	1.00			1.00		
1	1.23	(1.04	1.45)	1.18	(0.96	1.46)
2	1.23	(1.04	1.47)	1.23	(0.99	1.53)
≥3	2.43	(2.14	2.75)	2.73	(2.34	3.19)
**Type of treatment**						
Surgery only	1.00			1.00		
Surgery and chemo or radiotherapy	1.71	(1.53	1.92)	1.77	(1.55	2.02)
Chemo or radiotherapy only	4.41	(3.90	4.99)	4.86	(4.27	5.54)
**Type of hospital**						
Tertiary hospital	1.00			1.00		
General hospital	1.09	(1.00	1.19)	1.12	(1.01	1.23)

* HR: hazard ratio; CI: confidence interval; CCI: Charlson Comorbidity Index.

**Table 3:** Results of the subgroup analysis.

		All-Cause Mortality			All-Cause 5-Year Mortality		
		HR *	95% CI *		HR *	95% CI *	
**Disability severity**	**Disability**						
Mild	No	1.00			1.00		
	Yes	1.05	(0.92	1.19)	1.09	(0.95	1.26)
Severe	No	1.00			1.00		
	Yes	1.48	(1.24	1.76)	1.58	(1.29	1.93)

* HR: hazard ratio; CI: confidence interval.

## Data Availability

Data can be obtained for research purposes after application to the National Health Insurance Service.
